# Interface-Dependent Transport and Resistive Switching in TiO_2_ and TiO_2_:Co Nanotubes for Resistive Memories

**DOI:** 10.3390/nano16181179

**Published:** 2026-09-18

**Authors:** Y. Porras Ramírez, D. Laverde Lizarazo, Heiddy P. Quiroz, Jorge A. Calderón, A. Dussan

**Affiliations:** 1 Grupo de Materiales Nanoestructurados y sus Aplicaciones, Departamento de Física, Universidad Nacional de Colombia—Bogotá, Bogota 110001, Colombia; yporrasr@unal.edu.co (Y.P.R.); dlaverdel@unal.edu.co (D.L.L.); hpquirozg@unal.edu.co (H.P.Q.); jcalderonc5@ucentral.edu.co (J.A.C.); 2 Grupo de Investigación en Nanotecnología, Bioingeniería y Transferencia Tecnológica, Cluster in Convergent Sciences and Technologies, Universidad Central, Bogota 110311, Colombia

**Keywords:** non-volatile memory, O-DMS, resistive switching, spintronics

## Abstract

In this work, TiO_2_ and TiO_2_:Co nanotubes were fabricated via electrochemical anodization, using Ti (99.99% purity) and Ti/Co foils as the anode and cathode. Cobalt was deposited onto the Ti foils using the DC magnetron sputtering technique under a working pressure of 2.5 × 10^−2^ Torr. The resulting nanotubes exhibited wall nodes and an average length of 308.6 ± 13.07 nm for TiO_2_ and 92.63 ± 2.846 nm for TiO_2_:Co. The synthesized structures were characterized by X-ray diffraction (XRD), identifying anatase as the predominant phase, accompanied by an amorphous halo. Two types of MSM devices were fabricated to study bulk and surface conduction: a transverse configuration (TE/(TiO_2_, TiO_2_:Co)/Ti), with top electrodes (TE) of Al or Au, and a coplanar configuration (Al/TiO_2_/Al). Surface topography and surface-potential variations were investigated using Atomic Force Microscopy (AFM) and Kelvin Probe Force Microscopy (KPFM), respectively. The device behavior is mainly governed by the TE/TiO_2_ junction due to the absence of an energy barrier at the TiO_2_/Ti interface. All samples exhibit asymmetric I–V behavior with higher conduction under positive bias. The Au/TiO_2_/Ti device showed the lowest resistance among the Ti BE structures, displaying Schottky behavior with low reverse current leakage and a shift in the zero-current crossing depending on the scan direction. Barrier heights near the zero-current crossing, calculated via the thermionic emission model, were 0.91 eV and 0.70 eV for the reverse and forward directions, respectively. Ideality factors and series resistance exceeded 6.8 and 40 kΩ, respectively, suggesting additional transport mechanisms. In addition, magnetization as a function of the applied field was generated in the TiO_2_:Co nanotubes, evidencing their ferromagnetic-like behavior.

## 1. Introduction

In 1996, DARPA introduced the term ‘spintronics,’ derived from SPIN Transfer electronics, to spearhead the development of materials that use the degree of electron charge and spins of electrons [1]. Among spintronic materials, diluted magnetic semiconductors based on oxides (O-DMS) have emerged as a key component for integrating semiconducting and magnetic properties [2]. Notable properties of O-DMS include exhibiting ferromagnetic and antiferromagnetic behavior at room temperature [3], as well as the ability to control their resistive states (RS), making them promising candidates for non-volatile memory (NVM) applications [2].

TiO_2_, a versatile wide-bandgap semiconductor, has emerged as a promising candidate in spintronic applications due to its exceptional structural and electronic properties. When doped with transition metal elements such as Co, Mn, or Fe, TiO_2_ exhibits diluted magnetic semiconductor (DMS) behavior, enabling the integration of magnetic and semiconducting properties within a single material [3,4]. These dopants introduce localized magnetic moments and can mediate ferromagnetic or antiferromagnetic interactions, often observable at room temperature. Furthermore, the ability to tune its resistive switching (RS) properties through careful doping strategies positions TiO_2_ as an ideal material for the development of multifunctional devices, such as magnetic-resistive random-access memories (MRAM). Therefore, TiO_2_ and transition-metal-doped TiO_2_ have emerged as promising materials for spintronic applications owing to their attractive structural, electronic, and magnetic properties [2,5].

However, understanding and controlling the interplay between TiO_2_’s magnetic and resistive states remains a critical challenge, offering fertile ground for advancing spintronic technologies. The spintronic memristor, a magnetic memristor based on the spintronic effect of nanoelectrons, has emerged as a highly promising component in next-generation electronic and computational technologies [6,7]. By leveraging electron spin rather than solely relying on charge transport, this device overcomes several limitations of conventional memristors, particularly in terms of non-linearity arising from non-uniform doping and second-order effects [6,7,8]. It retains key advantages such as non-volatility, low power consumption, and ease of integration, while also exhibiting superior linearity, enhanced anti-interference performance, and improved reliability. These attributes make spintronic memristors highly attractive for applications in data storage, artificial intelligence, embedded systems [8], the Internet of Things (IoT) [9], and machine learning, where energy efficiency and stable performance are critical.

Recent advances in nanoscale semiconductor devices have demonstrated that electronic functionality cannot be understood solely from the intrinsic properties of the active material, since carrier density, interface quality, dimensionality, and device architecture can substantially modify charge transport. For instance, atomically thin semiconductor heterostructures have enabled efficient control of carrier transport and switching behavior through appropriate material and interface engineering [10]. Likewise, recent developments in field-effect devices based on low-dimensional materials have emphasized the critical role of electronic structure, charge transfer, surface interactions, and semiconductor interfaces in determining device performance [10]. These concepts are also relevant to transition-metal-oxide devices, where the electrical response can result from the combined effects of electrode/oxide interfaces and defect-assisted transport within the active layer. In TiO_2_-based systems, this interplay becomes particularly important because transition-metal incorporation can additionally modify the defect and electronic landscape, providing a route toward devices in which resistive-switching and magnetic responses coexist.

Furthermore, the continuously variable resistance of spintronic memristors, which is directly linked to charge variation over time, enables precise tunability and adaptability in real-time operational environments. As research continues to refine material properties and device architectures, the development of spintronic memristors is expected to pave the way for more efficient, high-performance memory and computing technologies, reinforcing their role in the advancement of spintronic and quantum-based devices. Therefore, this work presents the fabrication and characterization of TiO_2_ and TiO_2_:Co nanotubes, providing insights into their structural, electronic, and transport properties and highlighting their potential for spintronic and resistive-switching applications. The observed asymmetric I–V characteristics, Schottky-like interfacial behavior, and dependence on bias polarity indicate complex charge-transport mechanisms influenced by barrier heights, series resistance, and defect-related states. These findings contribute to a deeper understanding of transition-metal-doped TiO_2_ nanostructures and their role in advanced electronic devices, paving the way for further studies on their integration into next-generation memory and spintronic systems.

## 2. Materials and Methods

TiO_2_ and TiO_2_:Co nanotubes were fabricated via electrochemical anodization, using Ti foils (99.99% purity) with 50 µm and Ti/Co foils as the anode and Ti foils as the cathode. Cobalt was deposited onto the Ti foils by the DC magnetron sputtering technique using a Cobalt target of 99.995% purity, 50 W target power, under a working pressure of 2.5 × 10^−2^ Torr, and the deposition time was 30 min at room temperature. The electrochemical anodization process was carried out at room temperature in a solution containing 0.25 wt% ammonium fluoride (NH_4_F), 2 wt% distilled water, and 97.75% ethylene glycol. The total anodization time was 12 min, and the electrolyte was continuously stirred at 250 rpm throughout the entire anodization process. A square-wave voltage profile was applied (alternating voltage) characterized by 80 V for 1 min followed by 20 V during 5 min. The resulting nanotubes exhibited wall nodes and an average length of ~300 nm.

Al (99.99% of pure) and Au (99.99% of pure) top electrodes (TE) were vacuum-deposited onto the surface of the TiO_2_ and TiO_2_:Co via thermal evaporation. A tungsten boat was used at an evaporation temperature of 1473 K. Given the different mass and material properties, the conditions for Al and Au evaporation were as follows: 0.080 g of Al wire held for 15 s in a base pressure of $3.2\times 10^{-5}$ Torr; meanwhile, 0.150 g of Au pellets were held for 75 s under a vacuum of $7.4\times 10^{-4}$ Torr.

**Figure 1:**
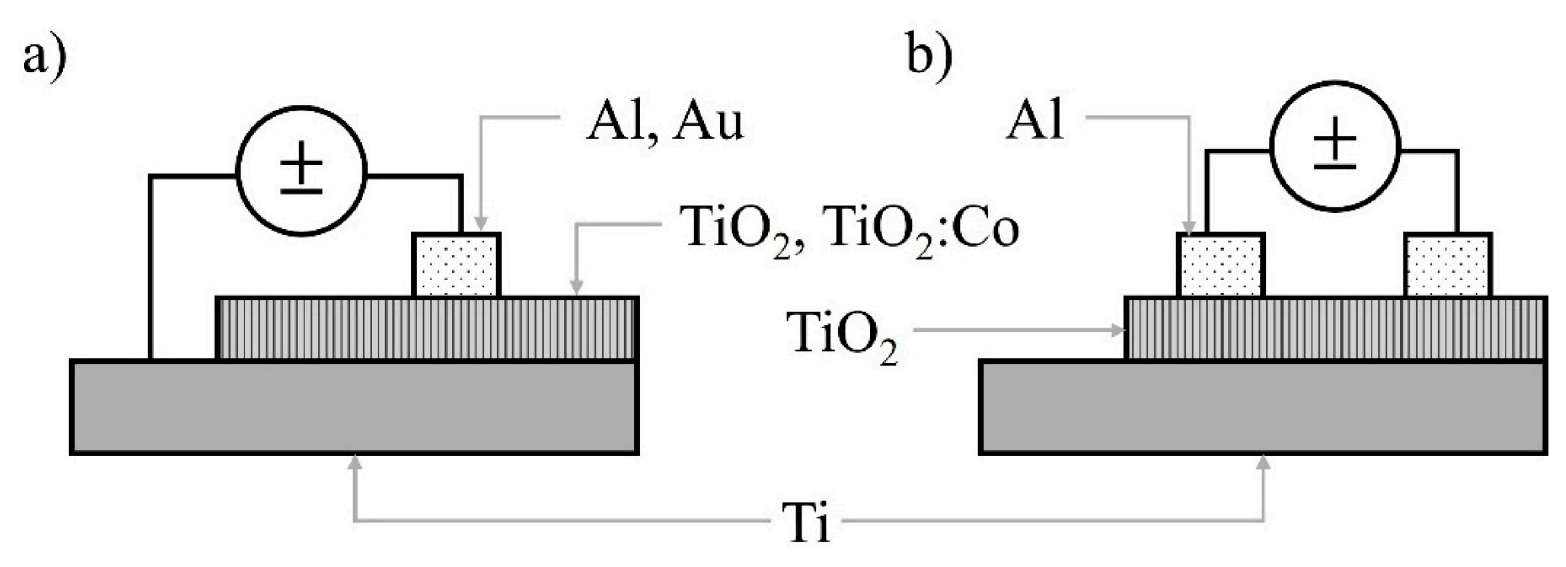
Schematic mechanism of (**a**) transversal and (**b**) coplanar structure of I–V measurements.

Two types of devices were fabricated to evaluate bulk and surface conduction behavior. The first type was a transversal structure, where the bottom electrode (BE) consisted of the Ti film serving as the substrate for the anodization process. The top electrode (TE) was either Au or Al, resulting in a TE/(TiO_2_, TiO_2_:Co)/Ti configuration. The second type was a coplanar structure with two aluminum electrodes (Al/TiO_2_/Al). Electrical measurements were carried out using a Keithley 2460 SourceMeter picoammeter (SEISA-Tektronix S.A. de C.V., Benito Juárez, México D.F.), with Remote LAN 1588 Interlock, under ambient temperature and atmospheric pressure (see Figure 1). The voltage was scanned in the sequence: 0 V → +3 V → 0 → −3 V → 0 V. Data analysis, including endurance measurements, was performed using computational tools.

The structural characterization of the synthesized samples was performed using X-ray diffraction (XRD) with a PANalytical X’Pert Pro diffractometer (PANalytical B. V., Almelo, The Neederland) equipped with a Cu-Kα radiation source (λ = 1.54 Å), operating at 40 kV and 40 mA, and an X’Celerator detector. The Rietveld refinement of the diffraction data was conducted using the X’Pert High Score Plus software (v2006). Morphological analysis was carried out using a Vega3 SB Scanning Electron Microscope - SEM (Vortex Company-Bruker, Brno, Czech Republic) with a tungsten source, an XFlash Detector 410 M (Vortex Company-Bruker, Brno, Czech Republic) and an acceleration voltage of 10 kV under high vacuum conditions (10^−6^ mbar). For SEM micrograph processing, ImageJ software (v1.54g) was used.

Magnetic measurements were performed at room temperature using a superconducting quantum interference device (SQUID) (Quantum Design MPMS 3 SQUID in Vibrating Sample Magnetometer (VSM) mode. The positive bias was defined as the current flowing from the BE to the TE. The films were evaluated by Atomic Force Microscopy (AFM) and Kelvin Probe Force Microscopy (KPFM) using a Cypher ES atomic force microscope (Asylum Research, Santa Barbara, CA, USA) operating device/analyzer in tapping mode.

## 3. Results

**Figure 2:**
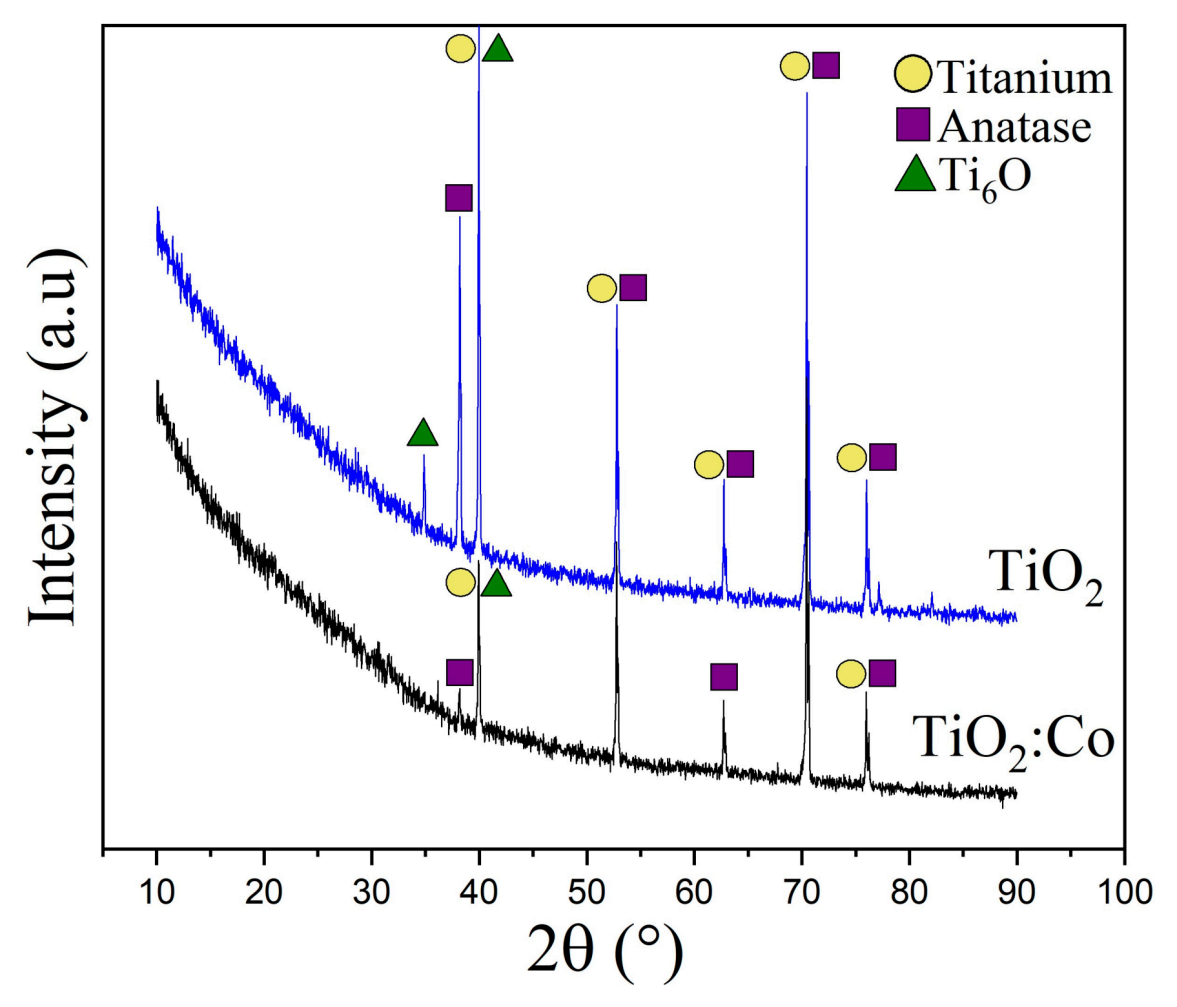
XRD patterns for TiO_2_ nanotubes (blue line) and TiO_2_:Co nanotubes (black line).

Figure 2 shows the XRD patterns for TiO_2_ and TiO_2_:Co nanotubes synthesized with alternating voltage at room temperature without annealing. As can be seen, the formation of an anatase phase is associated with the synthesis method and fabrication parameters, with titanium being the principal phase related to the substrate foil. Also, the presence of an amorphous halo without the formation of the Cobalt phases is clear. This is linked the Co low concentration of approximately 0.04 ± 0.01 wt% according to the EDXS measurements.

Importantly, the absence of Co-related diffraction peaks should not by itself be interpreted as direct evidence of substitutional incorporation of Co into TiO_2_, particularly considering the low Co content and the substantial amorphous contribution observed in the diffraction patterns. Therefore, the present results are compatible with Co being highly dispersed within the TiO_2_-based nanotubular structure, either occupying lattice-related sites or residing in locally disordered regions below the detection limit of XRD. Previous studies on Co-containing TiO_2_ systems have shown that Co incorporation can be accompanied by structural defects and oxygen-deficient environments [2,5].

Under anodization conditions where oxygen availability is limited, or where the rate of oxide formation exceeds the rate of oxygen incorporation, substoichiometric phases of the Ti-O system can be generated. In particular, the Ti_6_O phase forms in locally reducing environments characterized by a high density of oxygen vacancies. This phase represents a Ti-rich structure stabilized by the partial reduction of Ti^4+^ to lower oxidation states, such as Ti^3+^ or Ti^2+^ [11,12].

**Figure 3:**
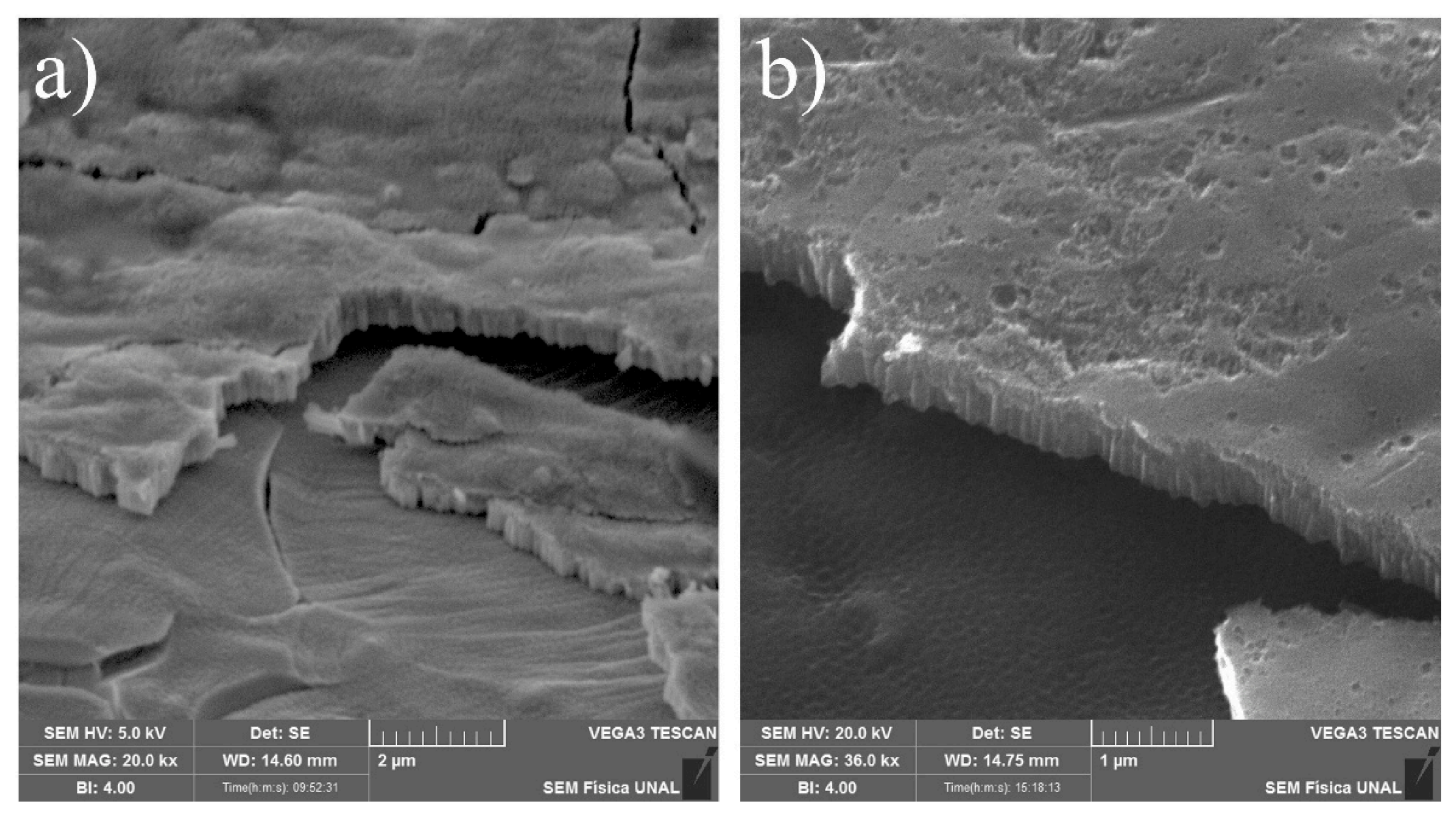
SEM micrographs of (**a**) TiO_2_ nanotubes and (**b**) TiO_2_:Co nanotubes.

Meanwhile, morphological characterization demonstrated the formation of nanotubes with an average length of 92.63 ± 2.846 nm for TiO_2_:Co samples, whilst the TiO_2_ samples show a mean length of 308.6 ± 13.07 nm. These measurements were taken through SEM micrographs (see Figure 3), and ImageJ software was used in the analysis of their sizes. Because the anodizing time is relatively short (12 min), close to the phase where the compact oxides are formed, and also because of the presence of Co, which generates other complexes ([TiF_6_]^2−^ and [CoF_6_]^3−^) [2] within the electrolyte during anodizing, the attack is more difficult, causing the observed difference in the size of the nanotubes in the samples.

**Figure 4:**
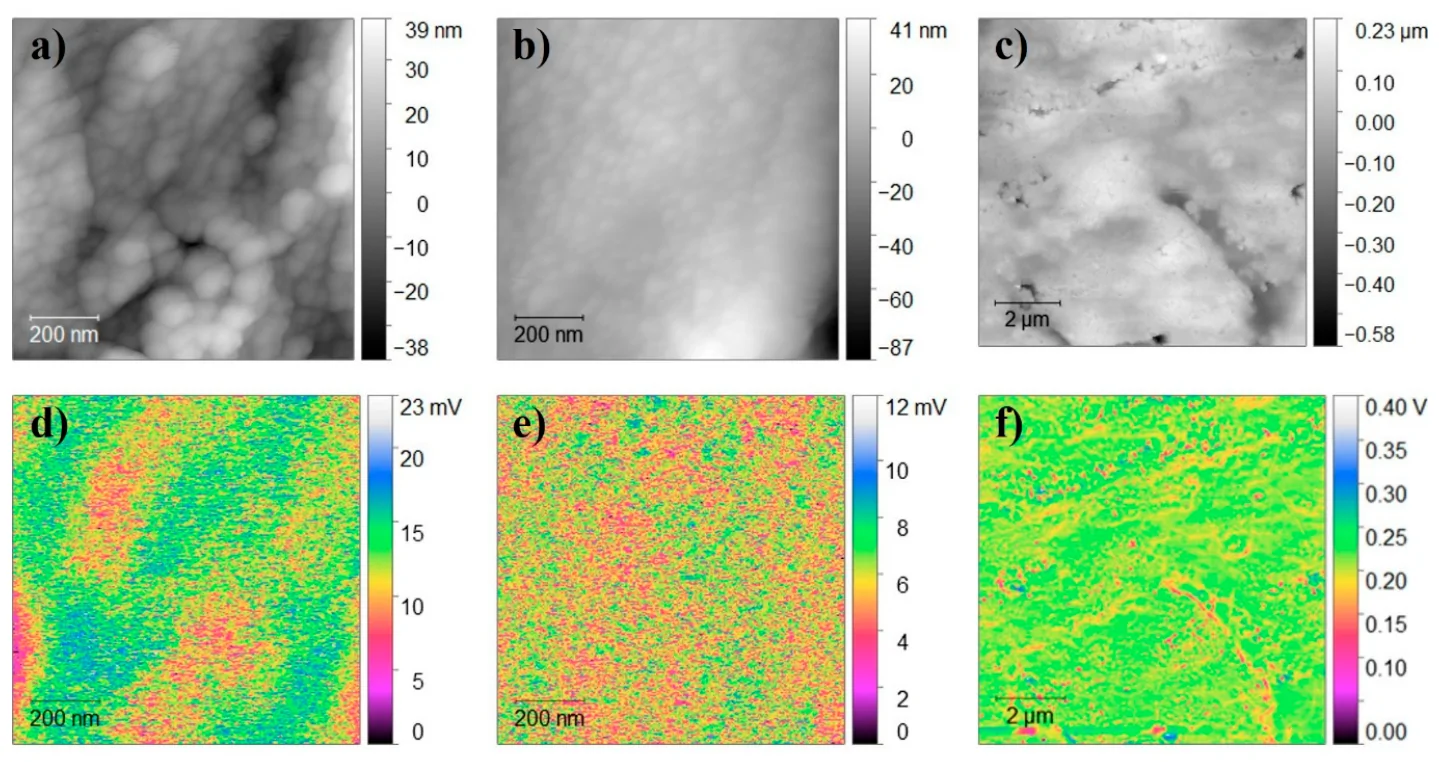
Topography (**a**–**c**) and Kelvin probe force microscopy (KPFM) surface potential maps (**d**–**f**) for TiO_2_-based samples. Images were acquired over scan areas of 1 μm^2^ for TiO_2_ (**a**,**d**), 1 μm^2^ for TiO_2_:Co (**b**,**e**), and 100 μm^2^ for TiO_2_ (**c**,**f**). KPFM maps are leveled to zero for comparison.

In addition, the analysis of the AFM micrographs reveals a textured surface characteristic of the nanotubular morphology of both TiO_2_ and TiO_2_:Co samples. However, the direct visualization of the inner diameters of the nanotubes is not feasible, as the AFM tip size is comparable to, or larger than, the inner diameters themselves. Consequently, the obtained topographic information primarily corresponds to the surrounding walls and external surface features of the nanotubes, rather than their hollow cores (see Figure 4a–c). This limitation must be considered when interpreting the surface morphology, as the AFM image reflects the convolution between the probe geometry and the sample topography. However, the KPFM maps present a slight correlation with the topography in areas deviating from the mean when the height range is about 0.8 μm (see Figure 4f). In a smaller height range (see Figure 4d,e), there is no evident correlation, which indicates the absence of charge aggregates on the surface of the nanotubes in either TiO_2_ or TiO_2_:Co sample.

**Figure 5:**
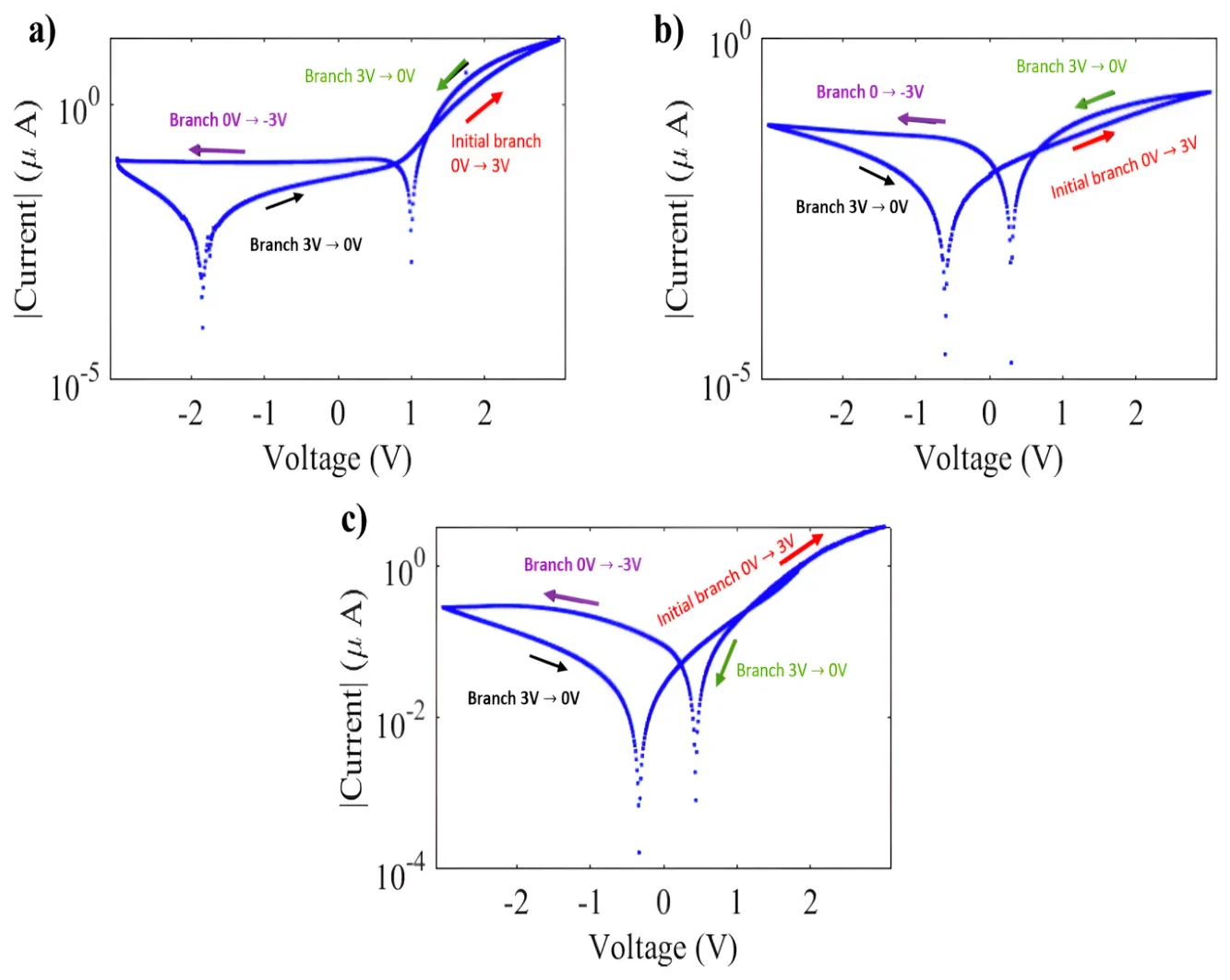
I–V curves of the samples with transversal structures: (**a**) Au/TiO_2_/Ti, (**b**) Al/TiO_2_/Ti, and (**c**) Al/TiO_2_:Co/Ti. The colored arrows indicate the voltage-sweep direction and identify the successive branches of the hysteresis cycle: red, initial sweep from 0 to +3 V; green, return sweep from +3 to 0 V; purple, sweep from 0 to −3 V; and black, return sweep from −3 to 0 V.

I–V characteristics of nanotubes obtained for the structures with Ti BE are shown in Figure 5 on a semi-logarithmic scale. The behavior is dominated by the TE and TiO_2_ junction, given the zero-junction energy barrier at the TiO2/Ti interface [13]. All samples present asymmetrical I–V behavior with higher conduction at positive bias.

The Au/TiO_2_/Ti sample (see Figure 5a) has the least resistance among the Ti BE structures analyzed, and it exhibits Schottky behavior with low reverse current leakage and a shift of the zero-current crossing depending on the scan direction, as previously observed in multiple Schottky junctions [14,15,16,17].

According to thermionic emission, the electrical parameters obtained from the I–V curves and the method developed by Cheung and Cheung are presented in Table 1. The barrier heights were calculated near zero current, where the curves do not deviate due to other transport mechanisms such as trap-assisted tunneling [16] and thermionic-field emission [14], or local potential barriers [14], as indicated by the high ideality factors and large values of series resistances. Nevertheless, the calculated barrier heights are in good agreement with the Au Work Function (5.1 eV) and TiO_2_ electron affinity (4.33 eV) reported in the literature [13,18], showing only small deviations attributable to the voltage scan direction.

Not all Al TE devices can be explained merely with a Schottky barrier, as there is also bipolar nonsymmetrical behavior (see Figure 5b,c), and a capacitive effect as a non-zero crossing point in the first quadrant [19]. Opposite to the increased conduction at positive bias, the hysteresis is stronger and more stable at negative bias. The asymmetry observed is consistent with the asymmetric layer stack.

**Figure 6:**
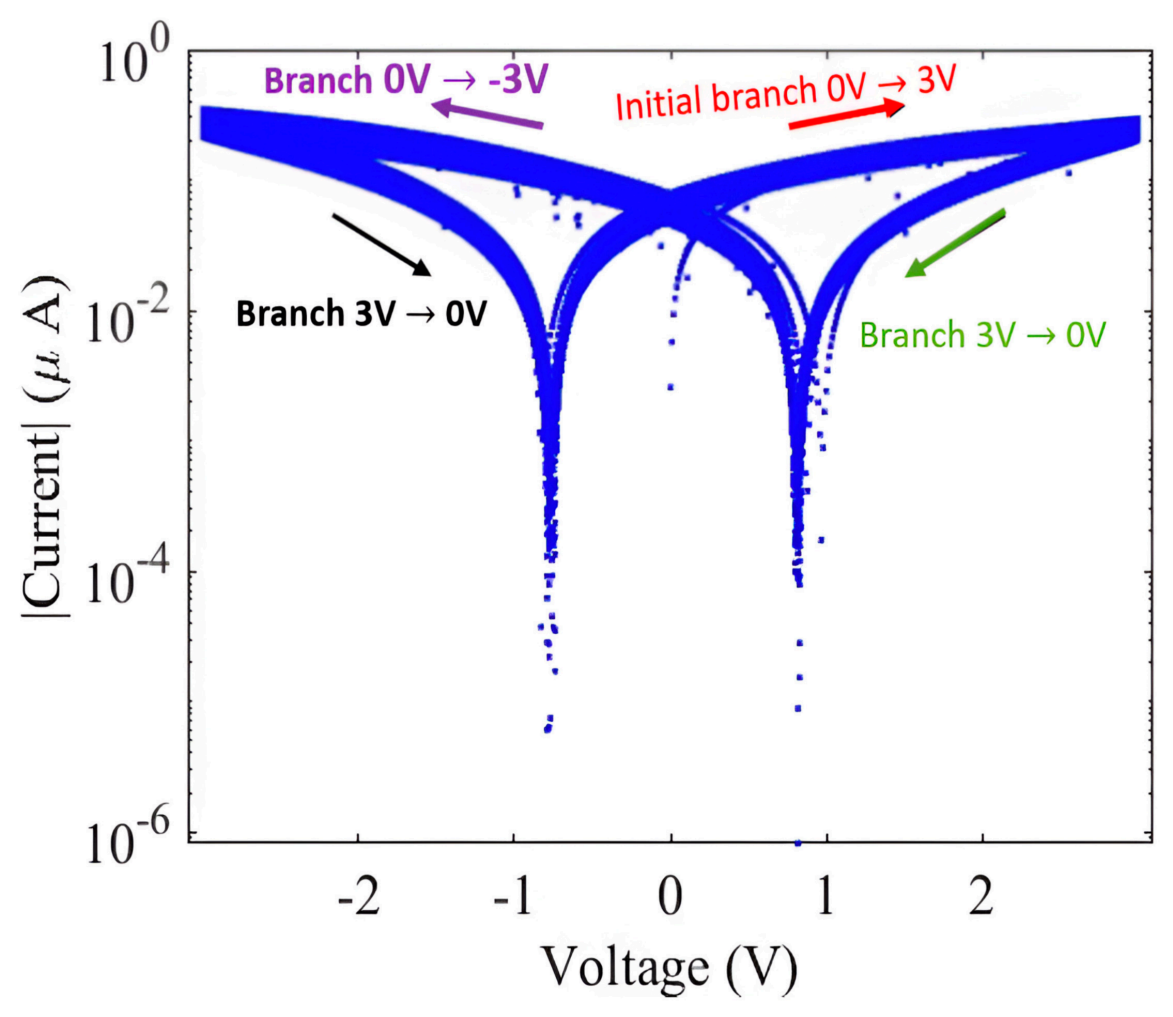
I–V curve for 50 cycles of the coplanar structure Al/TiO_2_/Al. The colored arrows indicate the voltage-sweep direction and identify the successive branches of the hysteresis cycle: red, initial sweep from 0 to +3 V; green, return sweep from +3 to 0 V; purple, sweep from 0 to −3 V; and black, return sweep from −3 to 0 V.

The coplanar device (see Figure 6) exhibits purely capacitive behavior. This configuration lacks a Ti bottom electrode and instead includes two Al top electrodes. In contrast, the transversal devices with Al TE (see Figure 5b,c) display both capacitive and memristive characteristics, where the capacitive response originates from the Al/TiO_2_ junction, while the memristive behavior is associated with the TiO_2_/Ti interface. Additionally, the Al/TiO_2_:Co junction exhibits a stronger capacitive response, as indicated by an increased crossing point compared to the TiO_2_-based structure.

Compared to the Au junctions, the Al junctions exhibit a more complex behavior that cannot be explained solely by work function and electron affinity differences. The presence of interface states between Al and the nanotubes—possibly formed during the evaporation process—may account for the observed parasitic capacitance. Although the deposition was carried out under high-vacuum conditions, this may not have been sufficient to prevent Al from reacting with residual particles. Additionally, the annihilation or incomplete formation of conduction filaments, leading to the creation of a barrier region, has also been reported as a possible origin of capacitive effects in memristive devices [19].

Although the Au/TiO_2_/Ti structure exhibits several features commonly associated with a Schottky contact, including rectifying behavior and barrier heights consistent with the Au work function and TiO_2_ electron affinity, the large ideality factors indicate a pronounced deviation from ideal thermionic-emission behavior. Therefore, the device should not be regarded as an ideal Schottky diode, but rather as a non-ideal Schottky-like junction in which interfacial and defect-assisted transport mechanisms contribute significantly to the current. The high values of n suggest that carrier transport is affected by interface states, local barrier-height inhomogeneities, trap-assisted tunneling, thermionic-field emission, and series-resistance effects [14,15,16]. These contributions are particularly plausible in the present nanotubular TiO_2_ system, where structural disorder, oxygen-deficient regions, and the large effective metal/oxide interfacial area can introduce a distribution of localized electronic states [15,16].

**Figure 7:**
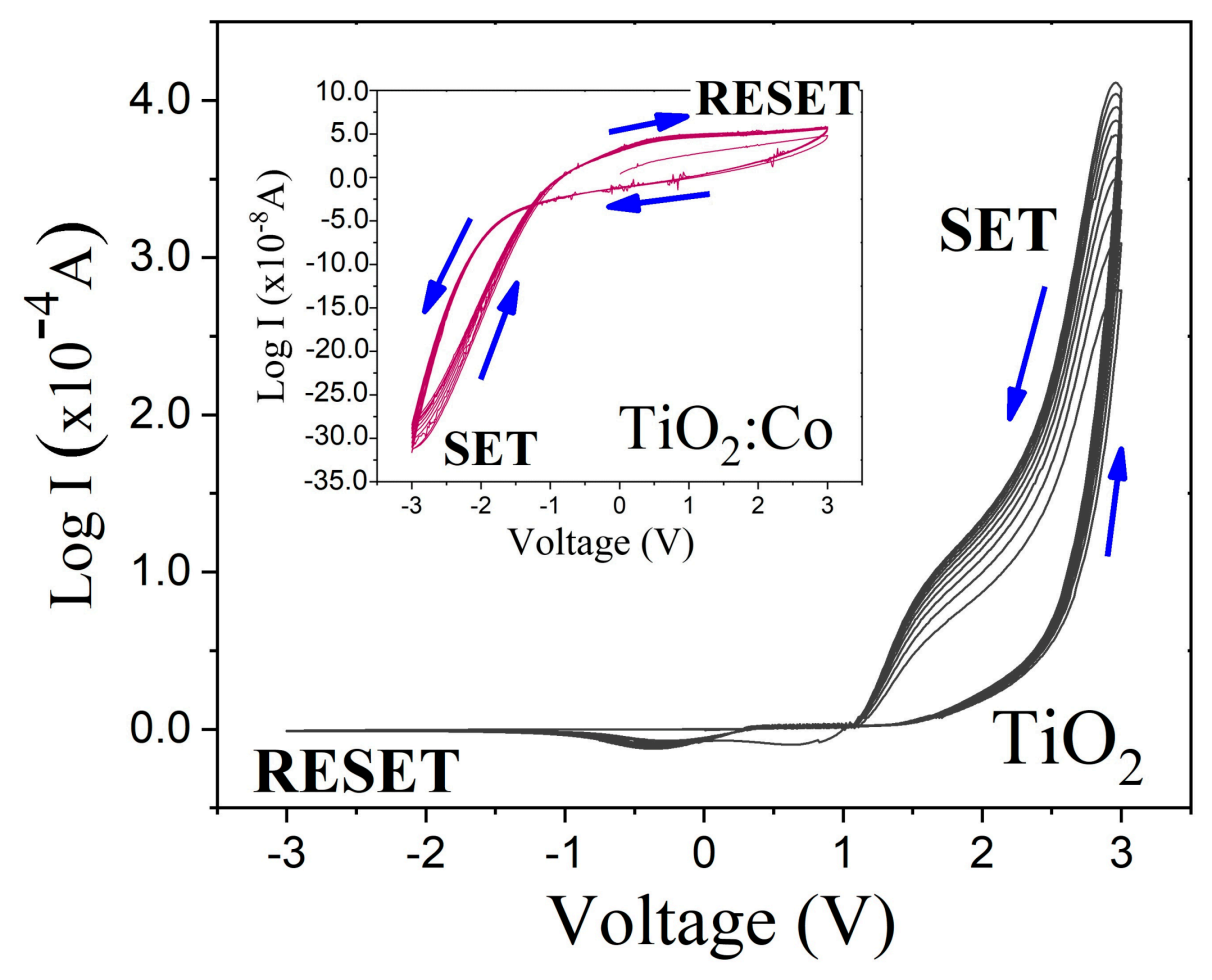
I–V curve for 10 cycles of the TiO_2_ nanotubes. Inset show I–V curve of TiO_2_:Co nanotubes.

In the case of the transversal I-V measurements, TiO_2_ nanotubes present a unipolar resistive switching behavior (see Figure 7). The SET state (High Resistance State (HRS)→Low Resistance State (LRS)) occurs in the positive region of the voltage, where there is an increase in current, and the RESET (LRS→ HRS) state occurs at negative voltages. In the inset of Figure 7, the I–V curves of the transversal section in the TiO_2_:Co nanotubes are presented. A bipolar resistive switching behavior can be observed, as demonstrated by the RESET state occurring in the positive voltage region and the SET state occurring in the negative voltage region. During the SET state, the conductive filament is formed within the dielectric layer, whereas in the RESET state, this filament is either ruptured or redistributed, leading to a transition back to the high-resistance state [12].

The size of the nanotubes plays a critical role in their memristive behavior. Thinner nanotube layers facilitate the formation of conductive filaments, enhancing the device’s switching performance. This filament formation underlies the resistive switching mechanism, enabling transitions between the high-resistance state (HRS) and the low-resistance state (LRS). The applied electric field and thermal effects induce the migration of free charge carriers—associated with amorphous TiO_2_ formation—and the generation of oxygen vacancies. When the density of these vacancies exceeds a critical threshold, they self-organize into a conductive filament. In TiO_2_-based resistive switching devices, the directionality and structural continuity of the nanotube walls further support and guide filament formation [2,20].

**Figure 8:**
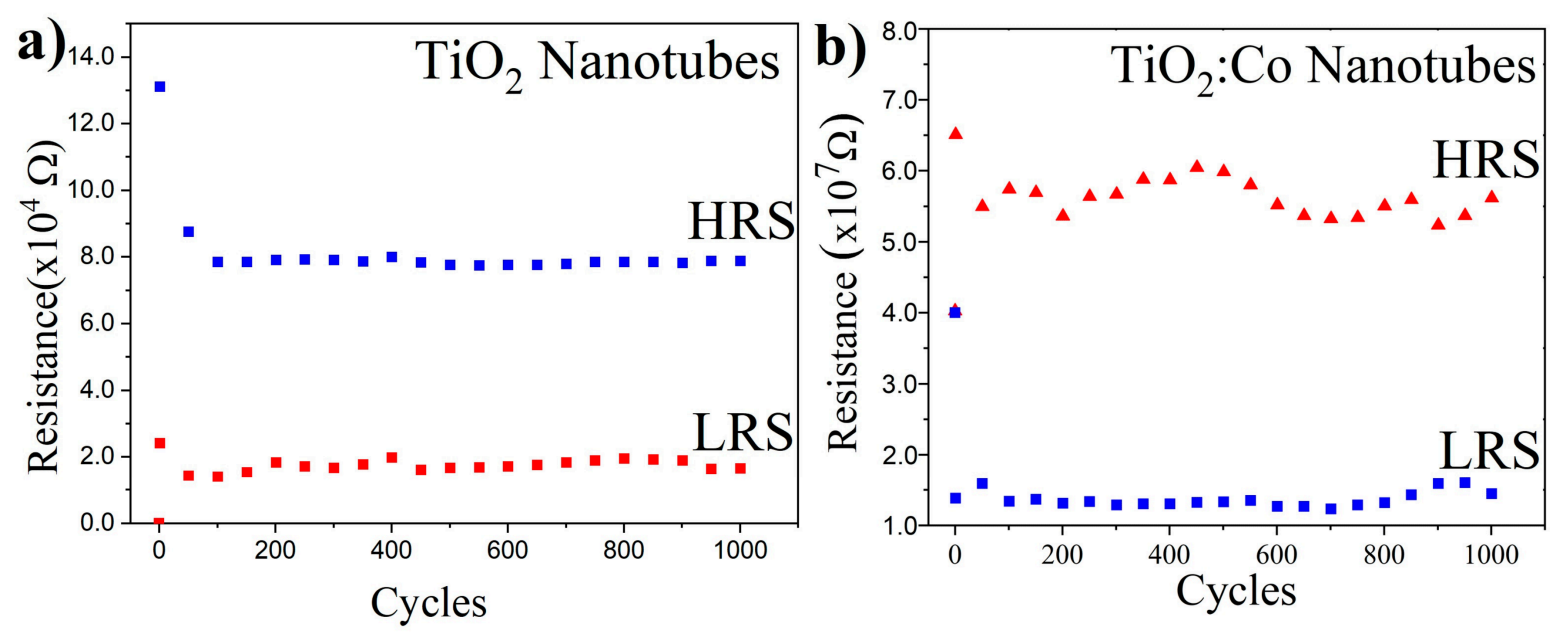
Endurance performance measurements for (**a**) TiO_2_ nanotubes (blue squares: HRS and red squares: LRS) and (**b**) TiO_2_:Co nanotubes (red triangles: HRS and blue squares: LRS).

Endurance performance measurements were also performed to assess the repeatability of the high-resistance state (HRS) and low-resistance state (LRS) after 1000 sweep cycles (1200 data points per cycle) for the TiO_2_ and TiO_2_:Co nanotubes (see Figure 8). The repeatability of the resistive states (HRS and LRS) was established in the SET region, under a positive bias of 2.0 V for TiO_2_ nanotubes and in the RESET region under a positive bias of 0.5 V for TiO_2_:Co nanotubes.

As shown in Figure 8a for TiO_2_ nanotubes, the first ~80 cycles exhibit the progressive formation of the conductive filament, followed by stabilization of both the high-resistance state (HRS) and low-resistance state (LRS), likely due to the increased mobility of free electrons [2]. A similar trend is observed in the endurance test for TiO_2_:Co nanotubes (Figure 8b), where conductive filament formation also occurs during the initial cycles, after which the switching behavior stabilizes. In this case, the LRS values remain relatively stable, while the HRS shows minor fluctuations. The incorporation of Co into the TiO_2_ matrix is believed to introduce defect states that enhance the operating voltage window and improve the endurance of the resistive switching process [2].

**Figure 9:**
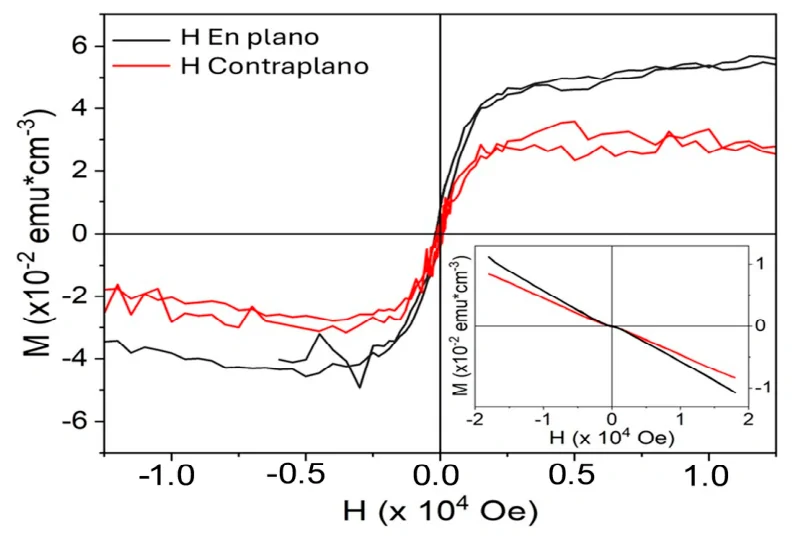
Magnetization (M) as a function of external magnetic field (H) curves. The black curves represent the measurements obtained with H applied parallel to the sample surface (in-plane, IP), whereas the red curves correspond to measurements performed with H applied perpendicular to the sample surface (out-of-plane, OP). The inset shows the corresponding magnetic response over an extended magnetic-field range.

Other studies have demonstrated that these materials can obtain magnetic properties that can aid in the resistive processes of memristor-type memory by incorporating transition elements into the semiconductor oxide matrix [5,21,22]. According to reports, one mechanism that supports the existence of dipole moments and contributes to ferromagnetic-like behavior is the incorporation of Co atoms into the semiconductor matrix. Figure 9 shows the magnetic behavior of the TiO_2_:Co nanotubes.

The magnetization (M) as a function of the applied magnetic field (H), measured with the field oriented in-plane (black line) and out-of-plane (red line), reveals the magnetic behavior of the TiO_2_:Co nanotubes. These measurements were corrected to remove the diamagnetic contributions from both the Ti substrate and the TiO_2_ matrix, thereby isolating the ferromagnetic-like response attributed to the system. This behavior is likely associated with weak exchange interactions between the magnetic moments of Co ions and the presence of structural defects within the TiO_2_ lattice [23,24]. The observed difference between the in-plane and out-of-plane magnetization curves suggests the presence of magnetocrystalline anisotropy [25], which may arise from the random spatial distribution of Co ions and their specific substitutional or interstitial positions within the semiconductor crystalline matrix. This observation is consistent with the Co concentration determined by EDXS analysis. The inset of Figure 9 shows the total magnetization, which includes the diamagnetic contribution from both the Ti foil substrate and the TiO_2_ semiconductor matrix.

## 4. Discussion

The structural, morphological, electrical, and magnetic results collectively demonstrate that the incorporation of Co into TiO_2_ nanotubes significantly modifies the electronic transport mechanisms while preserving the anatase crystalline structure. XRD measurements revealed that anatase remains the dominant phase after Co incorporation, whereas no secondary cobalt-related crystalline phases were detected within the sensitivity limits of the technique. The absence of detectable Co clusters suggests that cobalt is either substitutionally incorporated into the TiO_2_ lattice or distributed in highly dispersed defect-rich regions below the XRD detection threshold. Furthermore, the presence of an amorphous halo indicates a substantial degree of local structural disorder, which is expected to increase the density of defect states and oxygen-vacancy-related electronic levels. These defects play a crucial role in determining both the electrical conductivity and the magnetic response of oxide-based diluted magnetic semiconductors.

The SEM analysis revealed a pronounced reduction in nanotube length after cobalt incorporation, decreasing from approximately 309 nm in pure TiO_2_ to about 93 nm in TiO_2_:Co nanotubes. This behavior can be explained by the modification of the anodization kinetics induced by cobalt deposition prior to anodization. The formation of mixed fluoride complexes during electrochemical oxidation modifies the local dissolution and oxide growth rates, limiting nanotube elongation. Such morphological differences are expected to influence charge transport by modifying the effective conduction path and the density of structural defects distributed along the nanotube walls. Similar effects have been reported in transition-metal-doped TiO_2_ systems, where dopants alter both nanotube growth dynamics and defect formation processes.

AFM and KPFM measurements provide additional insight into the surface electronic properties. Although the nanotubular morphology was clearly observed in the topographic images, no significant localized surface potential variations were detected. This observation suggests a relatively homogeneous distribution of charge across the nanotube surface and indicates that the dominant transport mechanisms are likely governed by bulk defect states and metal–semiconductor interfaces rather than by localized surface charge accumulation. The absence of large potential fluctuations is also consistent with the low cobalt concentration determined by EDXS measurements, which would not be expected to generate strong electronic phase segregation.

The electrical measurements indicate that charge transport is primarily controlled by the top metal/TiO_2_ interface. The asymmetric current–voltage characteristics observed in both TiO_2_ and TiO_2_:Co devices are consistent with Schottky barrier formation at the top electrode, whereas the Ti/TiO_2_ interface behaves closer to an ohmic contact due to the favorable alignment of the Ti work function with the TiO_2_ conduction band. The lower resistance observed in Au/TiO_2_/Ti structures compared with Al/TiO_2_/Ti devices can be attributed to differences in work function and interface barrier formation. The extracted barrier heights near the zero-current crossing, together with the large ideality factors and series resistance values, indicate that pure thermionic emission cannot fully describe the transport behavior. Instead, conduction is likely assisted by defect-mediated mechanisms involving oxygen vacancies and localized electronic states distributed within the nanotube walls.

Additionally, the electrical response can therefore be interpreted as the combined contribution of interface-controlled injection and defect-assisted transport within the nanotube walls. The asymmetric I–V characteristics and the dependence on the top-electrode material indicate that carrier injection is strongly influenced by the metal/TiO_2_ interface. However, the large ideality factors and series resistances obtained for the Au/TiO_2_/Ti structure indicate that thermionic emission alone cannot account for the measured response. Once carriers are injected into the oxide, oxygen-deficient and structurally disordered regions can introduce localized electronic states that provide preferential pathways for charge transport [2,19,20].

A relevant difference between the TiO_2_ and TiO_2_:Co nanotube devices is the change from predominantly unipolar to bipolar resistive switching behavior. In the undoped TiO_2_ nanotubes, the SET and RESET processes are mainly governed by the magnitude of the applied electrical stimulus rather than its polarity. This behavior can be associated with the formation and disruption of conductive pathways involving electronic carriers supplied by intrinsic defect states, particularly those associated with oxygen-deficient regions within the oxide [2,20]. Once a sufficiently high electric field is applied, electrons can populate and percolate through these defect-related states, establishing a low-resistance conduction path. The subsequent disruption or partial deactivation of this path may therefore occur without requiring reversal of the electric-field direction, resulting in the observed unipolar response [20].

In contrast, Co-containing nanotubes exhibit bipolar switching, indicating that the conduction process becomes more sensitive to the direction of the applied electric field. The incorporation of Co is expected to modify the local electronic and defect landscape of TiO_2_ by introducing additional localized states and/or modifying the concentration and energetic distribution of intrinsic defects [2,5,20,22]. These changes can affect carrier trapping, detrapping, and the connectivity of defect-assisted conduction paths. Consequently, one voltage polarity may favor carrier injection and occupation of defect states, promoting the formation of a conductive pathway (SET), whereas reversal of the electric field may favor carrier detrapping and partial disruption of this pathway (RESET). In this framework, Co does not necessarily act as a mobile species during switching; rather, its role is proposed to be the modification of the electronic structure and defect-mediated transport of the TiO_2_ matrix, making the switching response more polarity-dependent [5,22].

The magnetic measurements further support the role of defects in determining the physical properties of the system. The observed ferromagnetic-like response at room temperature in TiO_2_:Co nanotubes is consistent with the behavior frequently reported in oxide-based diluted magnetic semiconductors [26]. Considering the low cobalt concentration and the absence of detectable cobalt-related crystalline phases, the magnetic response is likely associated with defect-assisted exchange interactions involving substitutional Co ions and oxygen-vacancy-related states. The coexistence of magnetic ordering and semiconducting transport makes TiO_2_:Co nanotubes particularly attractive for multifunctional devices where electrical and magnetic properties can be simultaneously exploited.

The magnetic response provides an additional indication that Co and defect-related electronic states modify the physical properties of the nanotubular system. After subtraction of the diamagnetic contributions of the Ti substrate and TiO_2_ matrix, the TiO_2_:Co nanotubes exhibit a weak ferromagnetic-like response at room temperature. Given the very low Co concentration detected by EDXS and the absence of Co-containing crystalline secondary phases detectable by XRD, the observed response cannot be straightforwardly assigned to a separate crystalline cobalt phase. Instead, it is consistent with defect-assisted magnetic interactions previously reported for transition-metal-containing TiO_2_ systems [1,5,20,22,23,24,25]. In these models, oxygen-deficient environments and the associated localized electronic states can participate in exchange interactions between spatially distributed magnetic moments, allowing magnetic correlations to develop even at relatively low dopant concentrations [22,23,24]. In particular, superexchange interactions have been discussed for Co-containing TiO_2_ [21], whereas defect-induced magnetic contributions in TiO_2_ have been extensively considered [23,24].

## 5. Conclusions

TiO_2_ and TiO_2_:Co nanotubes were successfully synthesized through electrochemical anodization using pure Ti and Co-doped Ti foils. The proposed method led to the formation of amorphous structures as well as the anatase phase containing oxygen vacancies, which facilitate the incorporation of cobalt into the TiO_2_ lattice without the emergence of binary phases. This approach enabled the development of diluted magnetic semiconductors with nanotubular morphology, with the average nanotube length decreasing from 308.6 ± 13.07 nm for TiO_2_ to 92.63 ± 2.846 nm for TiO_2_:Co. Furthermore, under anodization conditions with limited oxygen availability or where the oxide growth rate surpasses the oxygen incorporation rate, substoichiometric Ti–O phases such as Ti_6_O can be generated. Electrical response was evaluated, showing the absence of charge aggregates on the surface. I–V curves were measured to evaluate bulk and surface conduction behavior. Bulk conduction exhibited two distinct behaviors depending on the top electrode material: Au TE formed a Schottky barrier as expected, while Al electrodes introduced an additional capacitive component, attributed to the Al/TiO_2_ interface. Schottky-type transport was observed, with extracted barrier heights of approximately 0.70 eV in the forward direction and 0.91 eV in the reverse direction. The corresponding analysis yielded large ideality factors, ranging from approximately 6.8 to 16.68, together with series resistance values of about 40 kΩ and 70 kΩ for the forward and reverse branches, respectively. Surface conduction was evaluated using two Al electrodes, where no memristive effect was observed—only purely capacitive behavior was detected. The resistive switching behavior exhibited by TiO_2_ and TiO_2_:Co nanotubes is associated with the formation of a conductive filament, generated by the migration of free charge carriers under an applied electric field. This mechanism enables the transition between a high-resistance state (HRS) and a well-defined low-resistance state (LRS). Endurance tests performed at room temperature demonstrated the reproducibility of the HRS and LRS, with stable switching observed at a positive bias of 2.0 V for TiO_2_ and 0.5 V for TiO_2_:Co nanotubes. Finally, the M vs. H curves reveal that the magnetic response of the TiO_2_:Co nanotubes is primarily governed by magnetocrystalline anisotropy, which originates from the structural and compositional characteristics of the Co-doped system and is possibly related to defect-mediated ferromagnetism.

## Figures and Tables

**Table 1:** Schottky barrier parameters in the transversal structure Au/TiO_2_/Ti.

	Reverse Direction		Forward Direction	
Parameters	ln I–V	dV/dln(I)-V	ln I–V	dV/dln(I)-V
*n*	8.72	6.8	16.68	12.11
φ	0.91 eV	-	0.70 eV	-
Rs	-	70 kΩ	-	40 kΩ

## Data Availability

Data sets generated during the current study are available from the corresponding author upon reasonable request.

## Abbreviations

The following abbreviations are used in this manuscript:

KPFM	Kelvin Probe Force Microscopy
SEM	Scanning Electron Microscopy
XRD	X-Ray Diffraction
TE	Top Electrode

## References

[1] Bhardwaj P., Singh J., Verma V., Kumar R. (2025). Harnessing the duality of magnetism and conductivity: A review of oxide based dilute magnetic semiconductors. Phys. B Condens. Matter.

[2] Jaimes K.S., Quiroz H.P., Calderón J.A., Dussan A. (2024). Magnetic-resistive random access memories based on diluted Co-TiO_2_ nanotubes. Results Phys..

[3] Lee J., Song K., Shin K.-Y., Lee W. (2025). Improved resistive switching characteristics of Au/TiO_2_/Au memristors on PDMS substrates with pyramid arrays. Mater. Sci. Eng. B.

[4] Chen A. (2016). A review of emerging non-volatile memory (NVM) technologies and applications. Solid-State Electron..

[5] Quiroz H.P., Calderón J.A., Dussan A. (2020). Magnetic switching control in Co/TiO_2_ bilayer and TiO_2_:Co thin films for Magnetic-Resistive Random Access Memories (M-RRAM). J. Alloys Compd..

[6] Wen C., Zhang X., Guo X., Ru F., Quan S. (2024). Flame intensity sensor based on the resistive and memory properties of spintronic memristor. Sens. Actuators A Phys..

[7] Wen C., Xu L., Zha J., Zhou C. (2022). A novel nuclear radiation cumulant sensor based on spintronic memristor. Sens. Actuators A Phys..

[8] Paulo G., Sun K., Di Muccio G., Gubbiotti A., della Rocca B.M., Geng J., Maglia G., Chinappi M., Giacomello A. (2023). Hydrophobically gated memristive nanopores for neuromorphic applications. Nat. Commun..

[9] Lin Y., Zhang Q., Gao B., Tang J., Yao P., Li C., Huang S., Liu Z., Zhou Y., Liu Y. (2023). Uncertainty quantification via a memristor Bayesian deep neural network for risk-sensitive reinforcement learning. Nat. Mach. Intell..

[10] Nisar S., Dastgeer G., Shazad Z.M., Zulfiqar M.W., Rasheed A., Iqbal M.Z., Hussain K., Rabani I., Kim D., Irfan A. (2024). 2D Materials in Advanced Electronic Biosensors for Point-of-Care Devices. Adv. Sci..

[11] Kornilov I.I., Vavilova V.V., Fykin L.E., Ozerov R.P., Solov’ev S.P., Smirnov V.P. (1970). Neutron diffraction Investigation of Ordered Structures in the Titanium-Oxigen System. Metall. Trans..

[12] Bell C.N., Lee D.-C., Drexler M.N., Rouleau C.M., Sasaki K., Senanayake S.D., Williams M.D., Alamgir F.M. (2021). Substoichiometric Tuning of the Electronic Properties of Titania. Thin Solid Films.

[13] Hossein-Babaei F., Lajvardi M.M., Alaei-Sheini N. (2015). The energy barrier at noble metal/TiO_2_ junctions. Appl. Phys..

[14] Splith D., Müller S., von Wenckstern H., Grundmann M. (2021). Numerical Modeling of Schottky Barrier Diode Characteristics. Phys. Status Solidi A.

[15] Yu S., Zhang C., Yang P., Wu M., Sun Y., Li L. (2019). Effect of annealing temperature on structural and electrical properties of Al/Nb-doped TiO_2_ Schottky diodes on Pt-Si substrates. J. Mater. Sci. Mater. Electron..

[16] Zhang Y., Mauze A., Alema F., Osinsky A., Speck J.S. (2019). Near unity ideality factor for sidewall Schottky contacts on un-intentionally doped β-Ga_2_O_3_. Appl. Phys. Express.

[17] Zhao G., Yin Y., Peng Y., Yang W., Liu Y., Wang W., Zhou W., Tang D. (2019). Effect of hydrogen ions in the adsorbed water layer on the resistive switching properties of hexagonal WO_3_ nanowire. J. Appl. Phys..

[18] Endo R., Siriwardena H.D., Kondo A., Yamamoto C., Shimomura M. (2018). Structural and chemical analysis of TiO_2_ nanotube surface for dye-sensitized solar cells. Appl. Surf. Sci..

[19] Qingjiang L., Khiat A., Salaoru I., Papavassiliou C., Hui X., Prodromakis T. (2014). Memory Impedance in TiO_2_ based Metal-Insulator-Metal Devices. Sci. Rep..

[20] Quiroz H.P., Serrano J.E., Dussan A. (2020). Magnetic behavior and conductive wall switching in TiO_2_ and TiO_2_:Co self-organized nanotube arrays. J. Alloys Compd..

[21] Terán C.L., Calderón J.A., Quiroz H.P., Dussan A. (2021). Optical properties and bipolar resistive switching of ZnO thin films deposited via DC magnetron sputtering. Chin. J. Phys..

[22] Quiroz H.P., Galíndez E.F., Dussan A., Cardona-Rodriguez A., Ramirez J.G. (2021). Super-exchange interaction model in DMOs: Co doped TiO_2_ thin films. J. Mater. Sci..

[23] Stiller M. (2020). Defect Induced Magnetism in Titanium Dioxide. PhD Thesis.

[24] Kumar S., Kumari K., Kumar A., Koo B.H., Kumar R., Alvi P.A., Dalela S. (2023). 24-Magnetism of titanium dioxide. Defect-Induced Magnetism in Oxide Semiconductors.

[25] Hosseinpour P.M., Jiménez-Villacorta F., Liu J., Assaf B.A., McDonald I.J., Arena D., Heiman D., Menon L., Lewis L.H. (2018). Fe-incorporated TiO_2_ nanotube arrays: Electronic structure and magnetic response. Phys. Rev. B.

[26] Dussan A., Quiroz H.P., Calderón J.A. (2020). Nanomateriales que Revolucionan la Tecnología: Perspectivas y Aplicaciones en Espintrónica.

